# Predicting Long-Term Pain Resilience in Knee Osteoarthritis: An Osteoarthritis Initiative Nomogram

**DOI:** 10.3390/bioengineering13010096

**Published:** 2026-01-14

**Authors:** Ahmad Alkhatatbeh, Tariq Alkhatatbeh, Jiechen Chen, Hongjiang Chen, Jiankun Xu, Jun Hu

**Affiliations:** 1 Department of Orthopedics Surgery, The First Affiliated Hospital of Shantou University Medical College, Shantou 515041, China; 23alkhatatbeh@stu.edu.cn (A.A.); 15jcchen@stu.edu.cn (J.C.); chenhongjiang28@stu.edu.cn (H.C.); 2 Department of Joint Surgery, Center for Orthopedic Surgery, The Third Affiliated Hospital of Southern Medical University, Guangzhou 510630, China; tariq.alkhatatbeh@mail.nysy.com.cn; 3 Orthopedic Hospital of Guangdong Province, The Third Affiliated Hospital of Southern Medical University, Guangzhou 510630, China; 4 Guangdong Provincial Key Laboratory of Bone and Joint Degeneration Diseases, The Third Affiliated Hospital of Southern Medical University, Guangzhou 510630, China; 5 Department of Sports Orthopaedics, TUM University Hospital, Technical University of Munich, 81675 Munich, Germany; 6 Musculoskeletal Research Laboratory, Centre for Musculoskeletal Degeneration & Regeneration, Department of Orthopaedics & Traumatology, Faculty of Medicine, The Chinese University of Hong Kong, Hong Kong 999077, China

**Keywords:** knee osteoarthritis, pain resilience, prognostic model, WOMAC, nomogram, decision-curve analysis

## Abstract

Knee osteoarthritis prognostic tools often target structural progression or surgery and require imaging or biomarker inputs that are not routinely available. Using Osteoarthritis Initiative data, we developed a fully clinical nomogram to estimate both the probability of long-term pain non-resilience (clinically important worsening) and, by complement, maintenance of acceptable pain in radiographic knee osteoarthritis. We included participants with radiographic knee osteoarthritis and complete worst-knee WOMAC pain scores at baseline, 24 and 48 months; non-resilience was defined as a ≥9-point increase on the 0–100 WOMAC pain scale over 4 years. A six-predictor Firth logistic regression model (age, body mass index, Kellgren–Lawrence grade, baseline pain, 0–24-month pain change and Center for Epidemiologic Studies Depression Scale score) was fitted and translated into a point-based nomogram. Among 2365 eligible participants, 527 (22.3%) were non-resilient. The model showed good performance, with optimism-corrected AUC 0.74 and Brier score 0.15, and decision-curve analysis indicated positive net benefit versus treat-none across 1–15% thresholds and small gains versus treat-all. Early pain worsening and higher depressive symptoms were the strongest predictors of non-resilience. This six-variable, clinic-ready nomogram provides a simple, well-calibrated tool for prognostic counseling and risk stratification in radiographic knee osteoarthritis and requires external validation before wider clinical use.

## 1. Introduction

Knee osteoarthritis (OA) is common and characterized by chronic pain that drives care-seeking, functional limitation, and reduced quality of life. A large body of prognostic research in knee OA therefore focuses on predicting future pain, structural progression, or total knee arthroplasty (TKA) [1,2]. These efforts predominantly target adverse outcomes, such as onset or worsening of pain, frequent flares, or the need for surgery, rather than the complementary question of who remains well over time. OA is increasingly understood as a failure of the whole joint organ, involving cartilage, subchondral bone, synovium, ligaments, and periarticular muscles, and driven by both mechanical loading and systemic factors [2,3,4]. As a consequence, structural and symptomatic manifestations may be only loosely coupled at any single time point, and individuals follow distinct symptom trajectories over years rather than a single uniform course [1,3].

For patients and clinicians, the concept of “resilience,” defined as the sustained absence of clinically important deterioration, may be as important as progression. Recent work in the Osteoarthritis Initiative (OAI) has formalized sustained pain worsening using thresholds such as a ≥9-point increase on the 0–100 WOMAC pain scale [1]. These thresholds correspond to minimal or moderate clinically important changes supported by psychometric studies of WOMAC and related knee OA outcome instruments [5,6,7]. Framing stability as a positive outcome recognizes that maintaining acceptable pain and function is a key goal of long-term OA care. From a trajectory perspective, resilience corresponds to remaining on a low or stable pain track, whereas non-resilience reflects a transition to a persistently higher pain state; quantifying the risk of such transitions is particularly relevant when counseling patients about the long-term implications of current symptoms and early changes in pain [1,3,6].

State-of-the-art prediction models for knee OA often achieve moderate to strong discrimination but rely on specialized data. Models from the FNIH OAI Biomarkers Consortium reported AUCs around 0.76–0.77 for combined pain and radiographic progression, improving to approximately 0.86 when early changes in pain and imaging markers were added [8]. Deep learning methods using radiographs from the OAI have achieved AUCs of 0.77–0.81 for predicting the same ≥9-point WOMAC pain worsening outcome, particularly when image-based features are integrated with clinical variables [9]. Other recent nomograms for incident radiographic OA incorporate multiple serum protein biomarkers to reach AUCs in the 0.80–0.83 range [10]. Although powerful, such models may not be easily implemented in routine practice because they require MRI, specialized image processing pipelines, or biomarker assays that are not routinely available.

Consequently, there is a need for simple, clinic-ready tools that estimate an individual’s probability of maintaining pain resilience or, conversely, experiencing clinically important worsening, using information that is routinely collected in outpatient care. From a translational engineering perspective, such prognostic decision-support tools can be viewed as engineered clinical decision systems that map routinely observable clinical states to individualized risk estimates. Our primary aim was to develop and internally validate a pragmatic Clinical Pain Resilience Nomogram (CPRN) that estimates 4-year pain non-resilience in adults with radiographic knee OA using six pre-specified clinical and psychosocial predictors. This approach is intended to support practical clinical conversations about prognosis by translating baseline status and early symptom trajectory into an individualized risk estimate that can inform follow-up intensity, expectation setting, and consideration of targeted conservative strategies. A secondary aim was to characterize model performance, including discrimination, calibration, and potential clinical utility, to clarify how the CPRN could be used for prognostic counseling, shared decision-making, and risk stratification in routine care.

## 2. Materials and Methods

### 2.1. Study Design and Data Source

We conducted a secondary analysis of data from the Osteoarthritis Initiative (OAI), an ongoing multicenter longitudinal cohort of adults with or at risk of knee OA [11]. Participants were recruited at four U.S. clinical centers and followed annually with standardized clinical, radiographic, and questionnaire assessments. All data for this study were obtained from the public OAI repository. Reporting follows the TRIPOD and TRIPOD+AI statements [12,13]. We chose a 4-year horizon (baseline to 48 months) to capture durable symptom trajectories and restricted the analysis to knees with KL ≥ 2 to focus on definite radiographic OA. Data processing and cohort derivation were implemented through a fully scripted Python/R pipeline applied to the public clinical and radiographic releases, enhancing reproducibility and minimizing manual data handling.

The original OAI protocols were approved by institutional review boards at all participating sites, and all participants provided written informed consent. The present analysis used only de-identified, publicly available data and was considered exempt from additional review.

### 2.2. Participants and Cohort Assembly

We first identified 4796 OAI participants with worst-knee WOMAC pain scores available at baseline, 24 months, and 48 months (visits V00, V03, and V05). We required pain measurements at all three time points because the 48-month value defines the primary 4-year outcome, and the 24-month value is needed to compute early pain change (0 to 24 months), which was pre-specified as a key prognostic predictor. Accordingly, the CPRN should be interpreted as a 24-month landmark model that updates long-term risk using information available by the 24-month visit (baseline predictors plus early pain change). Knee-level clinical tables (including WOMAC pain and total scores, demographics, BMI, and KL grades) were linked to radiographic files and aggregated to the subject level by visit. KL grades were assigned using the Kellgren–Lawrence system [14]. For each visit, we defined “worse-knee” values as the maximum KL grade and maximum WOMAC pain score across the left and right knees; when both knees had equal values, the subject-level value was the same. This worst-knee approach reflects patient-level burden driven by the more severely affected knee and provides a reproducible way to summarize bilateral data for clinical risk prediction. This produced subject-level worst-knee WOMAC pain at baseline, 24 months, and 48 months plus baseline covariates; by design, all 4796 participants had non-missing worst-knee pain at these three time points.

**Figure 1:**
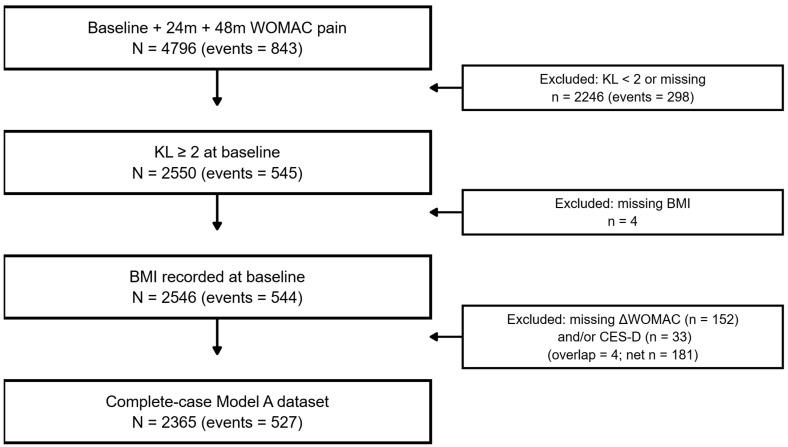
Cohort flow for the Clinical Pain Resilience Nomogram (CPRN) model. Flow of participants from 4796 Osteoarthritis Initiative subjects with complete 0/24/48-month Western Ontario and McMaster Universities Osteoarthritis Index (WOMAC) pain data to the Kellgren–Lawrence (KL) grade ≥ 2/body mass index (BMI)-complete and final complete-case cohorts, with non-resilience events shown at each step.

Figure 1 summarizes cohort refinement. Among the 4796 participants with complete pain follow-up, 843 (17.6%) met the ≥9-point non-resilience threshold on the 0–100 WOMAC scale. Restricting to radiographic knee OA (worse-knee KL ≥2) yielded 2550 individuals (545 [21.4%] non-resilient). Excluding four participants with missing BMI left 2546 participants (544 events). Finally, applying a complete-case requirement for the six pre-specified predictors (age, BMI, baseline KL grade, baseline WOMAC pain, early pain change 0–24 months, and baseline CES-D) removed 181 participants and resulted in a final analytic cohort of 2365 participants with 527 non-resilience events (22.3%). All subsequent analyses used this complete-case–cohort; missing data were not imputed. Baseline characteristics of participants included in the complete-case analytic cohort and those excluded due to missing predictor data are shown in Supplementary Table S1.

Worst-knee WOMAC pain scores and changes were harmonized across OAI releases using an automated scale-detection procedure that distinguished between 0–20 and 0–100 storage; details and threshold translation are described below.

### 2.3. Outcome Definition

The primary outcome was 4-year pain non-resilience, defined as clinically important worsening in worst-knee WOMAC pain between baseline and 48 months, operationalized as at least a 9-point increase on the 0–100 WOMAC pain scale [1,5,6,7,8]. For each participant, we computed Δ pain_0–48 (48-month minus baseline worst-knee WOMAC pain) on the scale in which the scores were stored. Because OAI releases store WOMAC pain on either a 0–20 Likert or 0–100 normalized scale, we inferred the underlying scale by inspecting the distribution of worst-knee values: if the maximum observed value was ≤25 points, we treated WOMAC pain as a 0–20 scale; otherwise, we treated it as a 0–100 scale.

We then translated the ≥9-point threshold from the 0–100 metric to the detected scale using proportional scaling (threshold = 9 × [scale/100]); for example, a 9-point increase on the 0–100 scale corresponds to a 1.8-point increase on the 0–20 scale. Non-resilience events were assigned when Δ pain_0–48 was greater than or equal to this scale-specific threshold. The model was parameterized to predict the probability of non-resilience; the probability of resilience was defined as 1 − P(non-resilience) [1,5,6,7,8].

In a pre-specified sensitivity analysis, we defined a broader composite outcome of clinically important worsening in either the WOMAC pain subscale or the WOMAC total score over 4 years. Using the same scale-detection and thresholding approach, composite non-resilience events were assigned when either Δ pain_0–48 or Δ total_0–48 exceeded the translated 9-point threshold.

### 2.4. Candidate Predictors

Six predictors were selected a priori based on clinical relevance and frequent use in knee OA prognostic models [2,8,9,10]. All were defined before examining outcome data. Predictors were derived from baseline assessments except early pain change, which incorporated the 24-month visit:

1. Age (years, continuous).
2. Body mass index (BMI) at baseline (kg/m^2^, continuous).
3. Baseline worse-knee KL grade (ordinal, grades 2–4), defined as the higher of the two knee grades at baseline (or the symptomatic knee if grades were equal).
4. Baseline WOMAC pain for the worse knee (higher scores indicating worse pain).
5. Early pain change, defined as Δ pain_0–24 (24-month minus baseline worst-knee WOMAC pain) on the stored scale.
6. Baseline depressive symptoms, measured by the Center for Epidemiologic Studies Depression Scale (CES-D; range 0–60; continuous).

For descriptive reporting and model interpretation, WOMAC pain and its change were summarized on the 0–20 Likert metric used in the AllClinical releases, with values converted to 0–100 when comparing with published minimal important change thresholds. All regression models were fit using 0–20 units for WOMAC pain and early pain change. Age, BMI, baseline WOMAC pain, early pain change, and CES-D were entered as continuous predictors, and worse-knee KL grade as an ordinal predictor (grades 2–4). All six predictors were forced into every model as a locked predictor set and modeled as linear terms on the log-odds scale without data-driven variable selection, interaction screening, or higher-order terms. Predictor retention did not depend on statistical significance because the objective was prognostic prediction rather than hypothesis testing.

### 2.5. Descriptive Analyses

We summarized cohort characteristics using means and standard deviations (SDs) for approximately normally distributed continuous variables, medians and interquartile ranges (IQRs) for skewed variables, and counts with percentages for categorical variables. Baseline characteristics were described overall and by non-resilience status. Non-resilience event counts and proportions were reported at each cohort refinement stage (full cohort, radiographic OA subset, BMI-complete subset, and final complete-case–cohort).

### 2.6. Model Development—Firth Logistic Regression (Primary Model)

The primary model was a Firth penalized logistic regression with the six pre-specified predictors and the 4-year pain non-resilience outcome [15,16]. We selected Firth penalization to reduce small-sample bias and to guard against unstable (potentially infinite) maximum-likelihood estimates that can arise under sparse predictor strata or (quasi-)separation in conventional logistic regression [15,16]. All predictors were entered as specified above; no data-driven variable selection, interaction terms, or non-linear transformations were considered. The model was fit in R (logistf), and we reported regression coefficients, odds ratios (ORs), and 95% confidence intervals (CIs) for each predictor.

Apparent performance in the complete-case–cohort was summarized using the area under the receiver operating characteristic curve (AUC) and the Brier score for predicted non-resilience. Calibration was assessed by fitting a logistic calibration model that regressed the observed outcome on the model’s linear predictor to obtain a calibration intercept and slope, and by comparing predicted and observed event rates across deciles of predicted risk.

Internal validation used 500 bootstrap resamples with out-of-bag evaluation. For each resample, we refit the Firth model and evaluated AUC and Brier score in the corresponding out-of-bag observations. The distribution of out-of-bag AUC and Brier values was summarized by the mean and 2.5th–97.5th percentiles to approximate expected out-of-sample performance of the CPRN model in similar populations [17,18].

### 2.7. Conventional Logistic Regression and Data Splitting

As a complementary analysis, we fit a standard (non-penalized) logistic regression model using the same six predictors and outcome. The complete-case dataset was randomly split into training, validation, and test sets in a 70/15/15 ratio, stratified by outcome. The training set was used to fit the logistic model, the validation set to estimate performance, and the held-out test set to provide an independent assessment of discrimination and overall accuracy. AUC and Brier scores were computed in the validation and test sets, and a test-set ROC curve was generated for visualization.

We also performed 500 bootstrap iterations with out-of-bag evaluation on the full complete-case dataset. In each iteration, we drew a bootstrap sample with replacement, refit the logistic model, and evaluated AUC and Brier score in the out-of-bag observations. The resulting distributions were summarized by their means and 2.5th–97.5th percentiles to provide an additional estimate of expected out-of-sample performance for the non-penalized model.

### 2.8. Decision-Curve Analysis Approach

Clinical utility of the CPRN model was evaluated using decision-curve analysis (DCA) [19,20]. For each threshold probability *p_t_*, net benefit was defined as:

$$Net\;benefit=TPR-FPR\times \frac{p_{t}}{1-p_{t}},$$

where TPR and FPR are the true-positive and false-positive rates at that threshold. Net benefit was computed for the CPRN model and compared with two default strategies: treating (or flagging) all patients as non-resilient and treating none. Threshold probabilities from 0.01 to 0.15 (1–15%) were chosen to represent low-to-moderate risk thresholds at which clinicians might consider intensified monitoring, counseling, or preventive interventions.

### 2.9. Sensitivity Analyses Plan

For the composite outcome sensitivity analysis (≥9-point increase in WOMAC pain or total score), we refit a standard logistic regression model using the same six predictors and reported sample size, number of events, AUC, and Brier score. A pre-specified secondary analysis attempted to restrict the cohort to participants in the OAI progression subcohort; however, after applying the KL ≥ 2, BMI, and complete-case requirements, too few participants remained to support a stable multivariable model.

### 2.10. Software

Data processing and conventional logistic regression analyses were performed in Python 3.12.3 using pandas 2.2.3, numpy 1.26.4, scikit-learn 1.6.1, statsmodels 0.14.6, and matplotlib 3.10.3. The primary Firth penalized logistic regression model, bootstrap internal validation, calibration analysis, nomogram construction, and decision-curve analysis were implemented in R 4.5.1 using logistf 1.26.1, rms 8.1.0, and pROC 1.19.0.1 (and supporting packages). For full reproducibility, the complete analysis pipeline is provided as (“Supplementary_Code_S1.zip”), including a README with run order and dependency lists.

## 3. Results

### 3.1. Cohort Flow and Baseline Characteristics

Participant flow is summarized as follows (Figure 1). Of 4796 OAI participants with complete baseline, 24-month, and 48-month WOMAC pain scores, 843 (17.6%) met the ≥9-point non-resilience threshold. Restricting to radiographic OA (worse-knee KL grade ≥ 2) yielded 2550 individuals (545 [21.4%] non-resilient); excluding four with missing BMI left 2546 (544 events). The complete-case–cohort for all six predictors comprised 2365 participants with 527 non-resilience events (22.3%) and was used for model development. A comparison of baseline characteristics for included versus excluded participants is provided in Supplementary Table S1.

In the KL ≥ 2/BMI-complete subset (*n* = 2546), mean age was 62.6 ± 9.0 years and mean BMI 29.6 ± 4.8 kg/m^2^; the median baseline KL grade was 2 (IQR 2–3). On the 0–20 WOMAC pain scale, median baseline pain was 3 (IQR 1–7), early pain change (0–24 months) was 0 (IQR −2 to 1), and CES-D was 5 (IQR 2–9) (Table 1).

In a descriptive comparison, the non-resilient group had BMI 30.2 ± 5.1 vs. 29.5 ± 4.7 kg/m^2^, median worse-knee KL grade 3 vs. 2, median baseline WOMAC pain 2 vs. 3 (0 to 20 scale), median delta WOMAC pain 0 to 24 of 1 vs. 0 (0 to 20 scale), and median CES-D score 5 vs. 4 (Table 1).

### 3.2. Firth Logistic Regression and Nomogram (Primary Model)

The Firth penalized logistic regression model included all six predictors in the complete-case–cohort (*n* = 2365; 527 events; Table 2). Estimated odds ratios per unit increase were: BMI 1.03 per kg/m^2^ (95% CI 1.01 to 1.05), worse-knee KL grade 1.30 per grade (95% CI 1.12 to 1.52), delta WOMAC pain 0 to 24 of 1.33 per 1-point increase on the 0 to 20 scale (95% CI 1.28 to 1.39), CES-D 1.02 per point (95% CI 1.01 to 1.04), baseline WOMAC pain 0.98 per point (95% CI 0.95 to 1.02), and age 1.00 per year (95% CI 0.98 to 1.01). Notably, the baseline WOMAC pain estimate was in the inverse direction (odds ratio below 1; Table 2), which may appear counterintuitive and is addressed in the Discussion.

**Figure 2:**
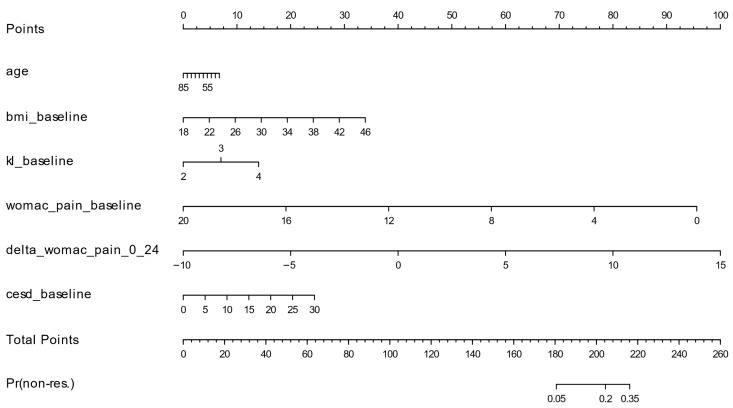
Clinical Pain Resilience Nomogram (CPRN). To use the nomogram: (1) For each predictor (age, body mass index [BMI], Kellgren–Lawrence [KL] grade, baseline Western Ontario and McMaster Universities Osteoarthritis Index [WOMAC] pain, 0–24-month change in WOMAC pain, and Center for Epidemiologic Studies Depression Scale [CES-D]), locate the patient’s value on its axis and draw a vertical line to the “Points” axis to read the points. (2) Sum the points across predictors to obtain “Total Points”. (3) Locate the total on the “Total Points” axis and draw a vertical line down to the predicted probability axis to obtain the predicted probability of 4-year pain non-resilience.

These estimates were translated into a nomogram (Figure 2) that assigns points to each predictor, sums them to a total points value, and maps total points to the predicted probability of non-resilience.

### 3.3. Discrimination, Calibration, and Overall Performance

**Figure 3:**
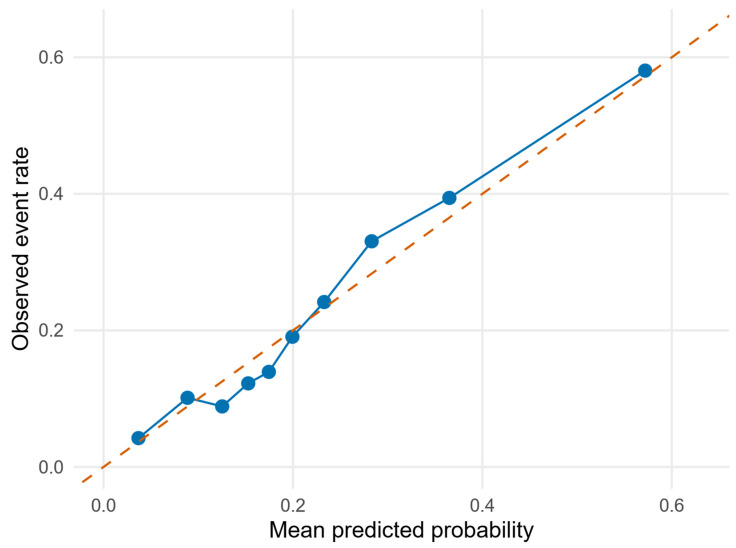
Calibration of the Clinical Pain Resilience Nomogram (CPRN) model. Decile-based calibration plot comparing observed and predicted 4-year non-resilience probabilities; the dashed 45° line denotes perfect calibration. The solid line shows the ROC curve of the model; the dashed diagonal line indicates chance-level discrimination.

Apparent performance in the complete-case–cohort was AUC 0.748 and Brier score 0.150. The apparent calibration intercept was ~0 and the calibration slope was ~1, and decile-based observed and predicted event rates are shown in Figure 3. Bootstrap internal validation with 500 resamples yielded a mean out-of-bag AUC of 0.742 (2.5th to 97.5th percentile 0.713 to 0.773) and mean out-of-bag Brier score of 0.151 (0.140 to 0.163).

### 3.4. Conventional Logistic Regression and Split-Sample Validation

**Figure 4:**
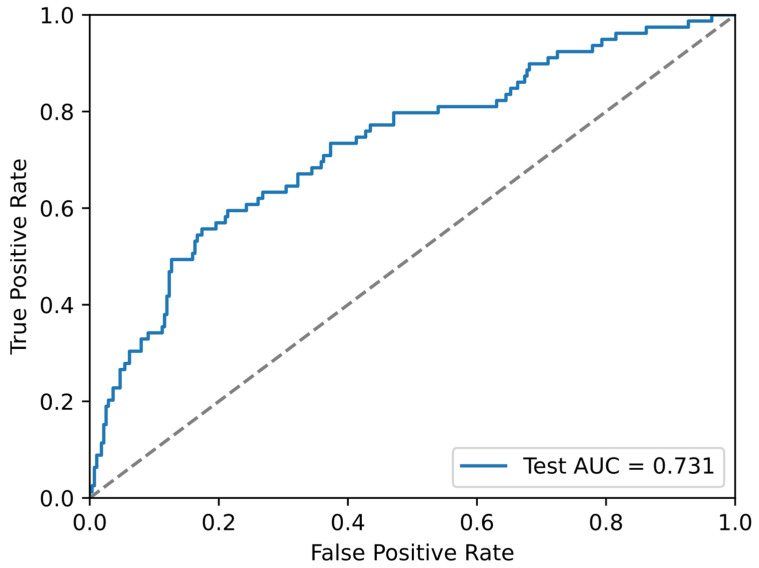
Test-set receiver operating characteristic (ROC) curve for the conventional logistic model. Receiver operating characteristic curve for the six-predictor logistic model in the 15% held-out test set from the 70/15/15 split. The solid line shows the ROC curve of the model; the dashed diagonal line indicates chance-level discrimination.

In a 70/15/15 train/validation/test split, the conventional logistic regression model achieved AUCs of 0.715 (validation) and 0.731 (test), with Brier scores of 0.156 and 0.150, respectively (Table 3; Figure 4). Bootstrap out-of-bag evaluation over 500 iterations yielded a mean AUC of 0.741 (95% CI 0.710 to 0.770) and mean Brier score of 0.151 (95% CI 0.140 to 0.164).

### 3.5. Decision-Curve Analysis

**Figure 5:**
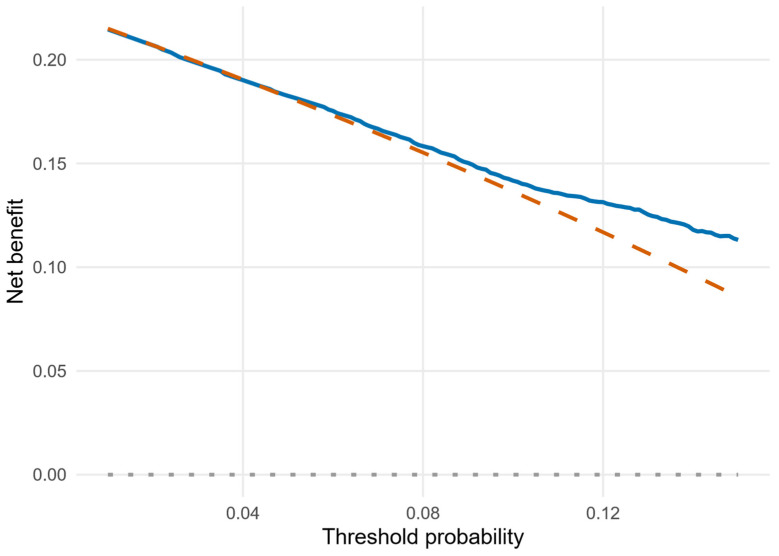
Decision-curve analysis for the Clinical Pain Resilience Nomogram (CPRN) model. Net benefit of the CPRN model versus treat-none and treat-all strategies across threshold probabilities from 1% to 15%. Solid line: CPRN model; dashed line: treat-all; dotted/flat line: treat-none (net benefit = 0).

As shown in Figure 5, decision-curve analysis compared the net benefit of the CPRN model with treat-none and treat-all strategies across threshold probabilities from 1% to 15%. At a 10% threshold, net benefit was 0.142 for CPRN and 0.136 for treat-all.

### 3.6. Sensitivity Analyses

With the broader composite outcome (≥9-point increase in either WOMAC pain or WOMAC total score over 4 years), 2365 participants were included and 611 (25.8%) experienced the composite non-resilience event, and the logistic model achieved an AUC of 0.740 and Brier score of 0.165 (Table 4).

The planned analysis restricted to the OAI progression subcohort had too few complete cases with KL ≥ 2 and all six predictors available to support a stable multivariable model. A separate progression-only CPRN model was therefore not fitted, and performance in that subset remains unknown.

## 4. Discussion

### 4.1. Principal Findings

In this secondary analysis of the OAI cohort, we developed and internally validated the Clinical Pain Resilience Nomogram (CPRN), a six-predictor model that estimates the probability of clinically important 4-year pain worsening in individuals with radiographic knee OA. Among participants with complete 0/24/48-month WOMAC pain data and KL ≥ 2 in the worse knee, about one in five met the non-resilience definition (≥9-point increase in WOMAC pain over 4 years). Using only routinely collected clinical and psychosocial variables, the CPRN model achieved an optimism-corrected AUC of 0.742 (95% CI 0.713–0.773) with good calibration across most of the risk spectrum.

Early pain change (0–24 months), baseline depressive symptoms, BMI, and KL grade were the most influential predictors. Early pain worsening was strongly associated with a higher probability of subsequent non-resilience. In the adjusted model, baseline WOMAC pain showed a modest inverse direction (odds ratio below 1), which may appear counterintuitive; this should be interpreted cautiously. Plausible explanations include ceiling effects in the WOMAC pain scale, regression to the mean, and the inclusion of early pain change (0 to 24 months) in the model, which may capture worsening risk more directly than baseline pain level alone. Higher CES-D scores were also associated with increased risk, and age contributed little incremental information beyond these factors.

### 4.2. Comparison with Previous Work

The CPRN model uses routinely collected clinical and psychosocial variables, and its discrimination is within the range reported for several more complex knee OA prediction models. FNIH OAI Biomarkers Consortium models for pain and radiographic progression reported AUCs of 0.76–0.77 using clinical and imaging predictors, improving to around 0.86 when early change in pain and additional biomarkers were incorporated [8]. Deep learning approaches applied to OAI radiographs have achieved AUCs of 0.77–0.81 for predicting the same ≥9-point WOMAC pain worsening threshold, particularly when image-based features are combined with clinical variables [9]. Other recent nomograms for incident radiographic OA that incorporate multiple serum protein biomarkers report AUCs in the 0.80–0.83 range [10]. Together, these studies highlight a trade-off between incremental gains in discrimination and reliance on specialized imaging or biomarker assays, whereas the CPRN model attains moderate discrimination using six readily available clinical and psychosocial predictors.

Direct numerical comparisons across studies should be interpreted cautiously because cohorts, outcome definitions, and validation strategies differ. Accordingly, we consider multiple dimensions of performance and validation when comparing models across studies.

AUC is only one aspect of model performance and should not be compared in isolation. Prior knee OA prognostic models vary substantially in endpoint definitions (for example structural progression, combined radiographic and pain progression, incident radiographic OA, or surgery), prediction horizons, and the type and rigor of validation reported. Some studies report primarily apparent discrimination, while others include internal validation and more complete calibration reporting. Because predicted probabilities are used for counseling and risk stratification, calibration metrics (such as calibration intercept, calibration slope, and Brier score) and clinical utility assessments (such as decision curve analysis) are also important when judging whether a model is likely to be reliable and useful in practice. For these reasons, we interpret cross-study AUC differences descriptively and emphasize that external validation, including calibration assessment in new populations, is required before broader implementation.

Nonetheless, selecting a ≥9-point change on the 0–100 WOMAC pain scale as the threshold for clinically important worsening is consistent with prior OAI-based analyses of pain progression [1,8] and psychometric work showing that changes of 9–12 points correspond to minimal important differences for knee OA pain [5,6,7]. The strong prognostic contribution of early pain trajectories aligns with longitudinal studies indicating that short-term pain worsening and fluctuations often precede sustained deterioration, whereas the independent effect of KL grade suggests that structural severity—although only modestly correlated with pain cross-sectionally—retains prognostic value for future symptom course [2,3]. The hypothesis that this reflects reduced “structural reserve” in more advanced disease, particularly among individuals with higher BMI, remains speculative and cannot be confirmed from the present observational data.

The association between depressive symptoms and pain non-resilience is consistent with work showing that depressive symptoms are dynamically and bidirectionally linked with knee pain and functional decline [21,22]. This is also consistent with systematic review evidence demonstrating an association between OA pain severity and symptoms of depression and anxiety [23]. These converging findings underscore the importance of psychosocial risk factors in symptom persistence and support integrating mental health screening and nonpharmacological interventions that address both pain and depressive symptoms within OA care pathways [24].

### 4.3. Clinical Implications

The CPRN model is best viewed as a tool for individualized prognostic counseling and risk stratification rather than a prescriptive rule for treatment selection. In routine knee OA care, decisions about major interventions (including referral and consideration of arthroplasty) integrate symptoms, function, radiographic severity, comorbidities, and patient preferences. The nomogram complements this process by quantifying a patient’s 4-year risk of clinically important pain worsening using variables that are typically available in standard visits (BMI, KL grade, baseline pain, early pain change, and depressive symptoms). A quantified risk estimate can help clinicians and patients anticipate potential symptom trajectories, align expectations, and motivate early, targeted non-surgical strategies such as structured exercise or physical activity programs, weight optimization, and psychosocial support, which are emphasized in contemporary hip and knee OA recommendations [25,26]. Evidence syntheses support weight-loss interventions for overweight or obese adults with knee OA as an approach to improve pain and function, reinforcing BMI as a clinically actionable risk factor [27]. In addition, systematic review evidence suggests psychological interventions, including cognitive behavior therapy, can improve OA-associated symptoms including pain and depressive symptoms [28].

Decision-curve analysis indicated modest but consistent net benefit across low-to-moderate threshold probabilities, with the CPRN model exceeding treat-none across 1% to 15% and slightly exceeding treat-all above approximately 2%. Although absolute gains were small, this pattern suggests that using the model to guide attention and monitoring may improve targeting compared with default strategies. In practice, clinicians could apply the nomogram at the 24-month follow-up visit to update risk and, when predicted risk exceeds a chosen threshold (for example 5% to 15%), consider more frequent reassessment, reinforcement of non-surgical therapies, and evaluation of psychosocial contributors to pain persistence and worsening.

### 4.4. Strengths and Limitations

Key strengths include the use of a large, well-characterized longitudinal cohort with standardized 4-year follow-up; a fully scripted pipeline to derive knee-level and subject-level cohorts, harmonize WOMAC scales, and construct prediction variables; and adherence to contemporary TRIPOD and TRIPOD+AI guidance with a prespecified six-predictor set and no data-driven variable selection [11,12]. We also examined robustness using complementary modeling strategies (Firth penalized and conventional logistic regression with bootstrap validation) and evaluated clinical utility using decision-curve analysis and a composite outcome sensitivity analysis.

Several limitations warrant consideration. First, although the number of events (527) was adequate relative to the number of predictors, the model has only been internally validated within the OAI cohort; external validation in independent populations, with calibration-in-the-large and slope updating as needed, is essential [29,30,31]. Second, complete-case analysis excluded participants with missing predictor data and may have introduced selection bias if missingness was not completely at random; future work should use multiple imputation [32]. Baseline characteristics of included and excluded participants are summarized in Supplementary Table S1. Third, we modeled all continuous predictors as linear terms without examining interactions or non-linear effects, which may obscure more complex relationships. Fourth, because early pain change (0 to 24 months) was included as a predictor, the CPRN is primarily intended for risk updating at a 24-month landmark visit rather than for use at a single baseline encounter. This design reflects the strong prognostic information contained in early symptom trajectories but may limit immediate baseline-only application; future work could develop and validate a baseline-only version and compare performance with the landmark model. Fifth, the CPRN model intentionally includes only clinical and psychosocial predictors and does not incorporate imaging features beyond baseline KL grade or biochemical biomarkers; this enhances feasibility but may cap discrimination relative to more complex multimodal models. Sixth, the inverse direction for baseline WOMAC pain should be interpreted cautiously and may reflect ceiling effects, regression to the mean, and mutual adjustment with early pain change (0 to 24 months) in this landmark model. Finally, because the analysis focused on individuals with radiographic OA (KL ≥ 2) and complete 4-year pain follow-up, estimated risks may not generalize to people with pre-radiographic OA, incomplete follow-up, or different care settings, and we could not assess performance in the OAI progression subcohort due to insufficient complete cases. As in all observational prognostic research, the associations should not be interpreted as causal, and the model is not intended to estimate the effects of modifying individual predictors.

### 4.5. Future Directions

Future work should prioritize external validation of the CPRN model in independent clinical cohorts, including community-based and specialty orthopedic populations [30,31]. Depending on differences in baseline risk and predictor distributions, calibration-in-the-large and model updating may be required, and studies should compare simple recalibration with more extensive revision [29,30,31]. Additional research could evaluate extended models that combine the six clinical predictors with quantitative imaging features, gait metrics, or serum biomarkers to quantify incremental gains in discrimination and clinical utility relative to the simple nomogram.

Another important direction is to adapt the concept of pain resilience to shorter time horizons (e.g., 1–2 years) and alternative, patient-centered outcomes such as sustained low pain or stable function. The existing scripted pipeline could be used to develop analogous models using 12-month pain change and trajectories of repeated CES-D measurements to support earlier identification of high-risk trajectories. Incorporating additional repeated psychosocial measures may further enhance dynamic risk prediction and help define thresholds that trigger specific management strategies in future prospective studies.

## 5. Conclusions

In this large longitudinal cohort of individuals with radiographic knee OA, clinically important 4-year pain worsening occurred in just over one in five participants, indicating that pain resilience is common but not guaranteed. We developed and internally validated the Clinical Pain Resilience Nomogram (CPRN), a six-predictor model based on routine clinical and psychosocial measures, which demonstrated moderate discrimination (optimism-corrected AUC 0.742; 95% CI 0.713–0.773) and good calibration for estimating the risk of pain non-resilience. The CPRN model is a pragmatic tool for prognostic counseling, shared decision-making, and prioritizing patients with early pain escalation or depressive symptoms for closer monitoring and targeted conservative interventions, but it requires external validation with calibration updating in diverse clinical settings before widespread implementation.

## Figures and Tables

**Table 1:** Baseline characteristics of the Kellgren–Lawrence (KL) grade ≥ 2/body mass index (BMI)-complete cohort.

Characteristic	Overall Cohort (*N* = 2546)	Resilient (*n* = 2002)	Non-Resilient (*n* = 544)
Age, years	62.6 (9.0)	62.7 (9.1)	62.2 (8.6)
BMI, kg/m^2^	29.6 (4.8)	29.5 (4.7)	30.2 (5.1)
KL grade (worse knee)	2.0 (2.0–3.0)	2.0 (2.0–3.0)	3.0 (2.0–3.0)
Baseline WOMAC pain (0–20)	3.0 (1.0–7.0)	3.0 (1.0–7.0)	2.0 (1.0–5.0)
ΔWOMAC pain 0–24 (0–20)	0.0 (−2.0–1.0)	0.0 (−2.0–1.0)	1.0 (0.0–3.0)
CES-D score	5.0 (2.0–9.0)	4.0 (2.0–9.0)	5.0 (2.0–10.0)

Values are mean (SD) for age and BMI and median (IQR) for WOMAC pain, ΔWOMAC pain 0–24, and CES-D, shown overall and by 4-year resilience status.

**Table 2:** Firth-penalized logistic regression model for 4-year non-resilience.

Predictor	Coefficient (β)	SE	Odds Ratio	95% CI for OR
Age (per year)	−0.004	0.006	1.00	0.98–1.01
BMI (per kg/m^2^)	0.029	0.011	1.03	1.01–1.05
KL grade (per grade)	0.266	0.079	1.30	1.12–1.52
Baseline WOMAC pain (0–20)	−0.017	0.017	0.98	0.95–1.02
ΔWOMAC pain 0–24 (0–20)	0.289	0.021	1.33	1.28–1.39
CES-D score	0.022	0.008	1.02	1.01–1.04

Regression coefficients (β), standard errors (SE), odds ratios (OR), and 95% confidence intervals (CI) for each of the six Clinical Pain Resilience Nomogram (CPRN) predictors. Abbreviations: β, regression coefficient; SE, standard error; OR, odds ratio; CI, confidence interval; CPRN, Clinical Pain Resilience Nomogram.

**Table 3:** Discrimination and overall performance of the Clinical Pain Resilience Nomogram (CPRN) and conventional logistic models.

Model/Dataset	AUC	95% CI (AUC)	Brier Score	95% CI (Brier)
CPRN (Firth), apparent	0.748	-	0.150	-
CPRN (Firth), bootstrap OOB	0.742	0.713–0.773	0.151	0.140–0.163
Conventional logistic, validation	0.715	-	0.156	-
Conventional logistic, test	0.731	-	0.150	-
Conventional logistic, bootstrap OOB	0.741	0.710–0.770	0.151	0.140–0.164

Apparent, split-sample, and bootstrap out-of-bag (OOB) area under the ROC curve (AUC) and Brier score for the Firth-penalized CPRN model and the conventional logistic model. Abbreviations: AUC, area under the curve; CI, confidence interval.

**Table 4:** Sensitivity analysis using a composite non-resilience outcome.

Analysis	N	Events (%)	AUC	Brier Score
Primary outcome	2365	527 (22.3%)	0.748	0.150
Alternative composite outcome	2365	611 (25.8%)	0.740	0.165

Sample size, events, AUC, and Brier score for the primary non-resilience definition and for a composite outcome (≥9-point increase in WOMAC pain or total score over 4 years).

## Data Availability

All data used in this analysis are publicly available from the Osteoarthritis Initiative (OAI) repository hosted by the NIMH Data Archive https://nda.nih.gov/oai/ (accessed on 5 September 2025). The complete analysis pipeline (Python and R scripts) is provided as (“Supplementary_Code_S1.zip”), including a README with run order and software dependencies.

## Supplementary Materials

The following supporting information can be downloaded at: https://www.mdpi.com/article/10.3390/bioengineering13010096/s1. Table S1. Baseline characteristics of participants included in the complete-case analytic cohort versus those excluded due to missing predictor data (within the Kellgren–Lawrence (KL) grade ≥2 and body mass index (BMI)-complete cohort).

## Abbreviations

The following abbreviations are used in this manuscript:

AUC	Area under the curve
BMI	Body mass index
CES-D	Center for Epidemiologic Studies Depression Scale
CI	Confidence interval
CPRN	Clinical Pain Resilience Nomogram
DCA	Decision-curve analysis
FNIH	Foundation for the National Institutes of Health
FPR	False-positive rate
ICMJE	International Committee of Medical Journal Editors
IQR	Interquartile range
KL	Kellgren–Lawrence
OA	Osteoarthritis
OAI	Osteoarthritis Initiative
OR	Odds ratio
ROC	Receiver operating characteristic
SD	Standard deviation
TKA	Total knee arthroplasty
TPR	True-positive rate
TRIPOD	Transparent Reporting of a Multivariable Prediction Model for Individual Prognosis or Diagnosis
WOMAC	Western Ontario and McMaster Universities Osteoarthritis Index
