# Supplementary material for: Predicting Long-Term Pain Resilience in Knee Osteoarthritis: An Osteoarthritis Initiative Nomogram
Source: Bioengineering (Basel). 2026 Jan 14;13(1):96. doi: 10.3390/bioengineering13010096 (PMC12837144; doi:10.3390/bioengineering13010096)
Supplement: Supplementary file 1 [file bioengineering-13-00096-s001.zip › Supplementary Table S1.pdf]

Supplementary Table S1. Baseline characteristics of participants included in the complete-case analytic cohort versus those excluded due to missing predictor data (within the Kellgren–Lawrence (KL) grade ⩾2 and body mass index (BMI)-complete cohort).

Variable	Included (complete-case) (N=2365)	Excluded (missing predictors/outcome) (N=181)	Missing in eligible cohort	p_value
Age, years	62.71 (8.98)	60.85 (9.12)	0 (0.0%)	0.009
BMI, kg/m^2	29.53 (4.79)	30.81 (4.78)	0 (0.0%)	<0.001
WOMAC pain (baseline)	3.98 (3.88)	5.72 (4.57)	0 (0.0%)	<0.001
CES-D (baseline)	6.57 (6.95)	8.74 (8.38)	33 (1.3%)	0.002
KL grade 2	1264 (53.4%)	99 (54.7%)	0 (0.0%)	0.947
KL grade 3	829 (35.1%)	62 (34.3%)		
KL grade 4	272 (11.5%)	20 (11.0%)		
Sex 1: Male	999 (42.2%)	73 (40.3%)	0 (0.0%)	0.672
Sex 2: Female	1366 (57.8%)	108 (59.7%)		
Race 0: Other	41 (1.7%)	1 (0.6%)	0 (0.0%)	<0.001
Race 1: White	1867 (78.9%)	96 (53.0%)		
Race 2: Black	442 (18.7%)	77 (42.5%)		
Race 3: Asian	14 (0.6%)	7 (3.9%)		
Race: Missing/Unknown	1 (<0.1%)	0 (0.0%)		

P-values for categorical variables are from overall chi-square or Fisher's exact tests across categories.
